# Patients as teachers: a within-subjects randomized pilot experiment of patient-led online learning modules for health professionals

**DOI:** 10.1186/s12909-024-05473-4

**Published:** 2024-05-10

**Authors:** Ruth Ndjaboue, Caroline Beaudoin, Sandrine Comeau, Anne Dagnault, Maman Joyce Dogba, Sarah Numainville, Charles Racine, Sharon Straus, Marie-Claude Tremblay, Holly O. Witteman

**Affiliations:** 1 Université de Sherbrooke, Sherbrooke, Canada; 2 Université Laval, Québec City, Canada; 3 University of Toronto, Toronto, Canada

**Keywords:** Equity, Diversity, Inclusion, Patient partnership, Online learning

## Abstract

**Purpose:**

Many health professions education programs involve people with lived experience as expert speakers. Such presentations may help learners better understand the realities of living with chronic illness or experiencing an acute health problem. However, lectures from only one or a small number of people may not adequately illustrate the perspectives and experiences of a diverse patient cohort. Additionally, logistical constraints such as public health restrictions or travel barriers may impede in-person presentations, particularly among people who have more restrictions on their time. Health professions education programs may benefit from understanding the potential effects of online patient-led presentations with a diverse set of speakers. We aimed to explore whether patient-led online learning modules about diabetes care would influence learners’ responses to clinical scenarios and to collect learners’ feedback about the modules.

**Method:**

This within-subjects randomized experiment involved 26 third-year medical students at Université Laval in Quebec, Canada. Participation in the experiment was an optional component within a required course. Prior to the intervention, participating learners responded to three clinical scenarios randomly selected from a set of six such scenarios. Each participant responded to the other three scenarios after the intervention. The intervention consisted of patient-led online learning modules incorporating segments of narratives from 21 patient partners (11 racialized or Indigenous) describing why and how clinicians could provide patient-centered care. Working with clinical teachers and psychometric experts, we developed a scoring grid based on the biopsychosocial model and set 0.6 as a passing score. Independent evaluators, blinded to whether each response was collected before or after the intervention, then scored learners’ responses to scenarios using the grid. We used Fisher’s Exact test to compare proportions of passing scores before and after the intervention.

**Results:**

Learners’ overall percentage of passing scores prior to the intervention was 66%. Following the intervention, the percentage of passing scores was 76% (*p* = 0.002). Overall, learners expressed appreciation and other positive feedback regarding the patient-led online learning modules.

**Discussion:**

Findings from this experiment suggest that learners can learn to provide better patient-centered care by watching patient-led online learning modules created in collaboration with a diversity of patient partners.

**Supplementary Information:**

The online version contains supplementary material available at 10.1186/s12909-024-05473-4.

## Introduction

Previous research has shown that people living with chronic disease (hereafter “patients”) can offer valuable insights for improving health professions education because patients have unique expertise concerning the impact of a condition and its care on their own lives [1–4]. Sharing such expertise in the course of health professions education may help foster high-quality and personalized care [5–7]. Accordingly, many educational settings organize presentations in which patients share their lived experiences and expertise. Although in-person presentations offer the potential benefits of allowing direct interactions between patients and health professions students (hereafter “learners”), in-person involvement can also be challenged by constraints such as patients’ hospitalization, timing, geographical distance, family responsibilities, and, with the advent of the Covid-19 pandemic, public health restrictions. In-person sessions may make it especially difficult to ensure learners can learn from a wide variety of patients from diverse backgrounds; for example, patients who live far from the medical school (for example, in a remote Indigenous community), who have transportation challenges (for example, lack of easy access to a personal vehicle), or who have less flexibility in their lives (for example, due to family or work responsibilities.)

The experiences of minoritized patients may appear less frequently in health professions curricula simply because of the patients’ minority status. Yet, these experiences are arguably more important for learners to hear given that people who are minoritized are more likely to have negative healthcare experiences [7–10]. This may be especially true for learners who are members of majority groups and who may therefore have had fewer opportunities to become aware of such negative patterns within healthcare [11, 12]. The lessons offered through the stories of people who are minoritized are crucial to increase cultural safety and anti-racism in healthcare [13–15]. 

Our previous work suggested that pre-recorded, patient-led videos may offer a potential alternative to in-person presentations and more easily integrate perspectives from people of diverse backgrounds [16]. The purpose of this study was to evaluate whether patient-led pre-recorded online learning modules on diabetes care can influence learners’ responses to clinical scenarios, related questions, and to collect learners’ feedback about videos.

## Methods

### Prior work

We previously video-recorded interviews with 21 people, including 11 self-identified Indigenous or racialized people, living with various types of diabetes, either their own diabetes or that of a child or spouse. The videos focused on how and why to improve the quality of healthcare offered to patients and caregivers, including through responses to the question, “What knowledge, wisdom, or advice do you want to share with health professionals who care for people living with diabetes like you or your loved one to help them provide better care?” and, if needed, the prompt, “What should they do or not do, or say or not say to provide better care?” [17] These 21 patient partners had been identified and invited to be part of a national research network due to their ability to bring meaningful experiential expertise of diabetes care, while reflecting on self-experiences and how these might or might not reflect the experiences of other patients [18]. Participants were proficient in at least one of French or English and were welcome to record their videos in French or English, and to include statements in other languages and explain their statement in French or English as they deemed appropriate. Three bilingual researchers (RN, SCD, HW), including one living with diabetes (HW), analyzed narratives and synthesized information into themes and sub-themes using inductive framework analysis [19]. Throughout the process, the research team gave everyone appearing in the videos the opportunity to revise and contribute to the online learning modules, and to have sections of their videos changed or removed if they wished. With the assistance of a specialist from the Faculty of Medicine’s audiovisual department who cut the recorded interviews and edited them into learning modules, we ultimately produced patient-led online learning modules in French (the language of the educational institution of the present pilot study) incorporating segments of 19 of initial 21 narratives organized thematically. Modules included subtitles in French during segments where patients speak in English. In the online learning modules, patient partners said things like, “Check in on the parents because it is hard on the parents too,” “There is a lot of shame in diabetes. It’s one of the only chronic illnesses where the people who live with it are constantly blamed,” and, “As an Indigenous man, I expect health professionals to speak to me as an equal.” [20] The total viewing time for all modules was 45 min.

To assess whether and how our patient-led learning modules aligned with the academic and clinical expectations of the participating institution, two resident physicians (BB, FFT) independently analyzed modules to check the alignment of patients’ perspectives with CanMEDS, the internationally recognized competency-based assessment framework, which outlines six expected roles of future physicians [21]. The residents met regularly to compare their ratings, discuss any disagreements, and recode sections as they refined their interpretations. The kappa coefficient reflecting their inter-rater agreement was 0.93. The residents’ independent coding aligned with our previous findings that patients’ suggestions focused heavily on communication in health care [20]. 

### Study design

This study reports a within-subjects double-blinded pilot experiment. In within-subjects experiments, the experimental and control data are collected on the same people. Within-subjects designs randomly assign study participants to multiple sequences while making each participant their own control for data comparison. Such designs offer an efficient way to detect effects of interventions using smaller sample sizes than would be required for a between-subjects experiment [22]. In keeping with the goal of rigorously studying health professions pedagogy, they also provide a way to use randomized designs in real-world training contexts while remaining within accreditation constraints stipulating that all learners must have access to the same educational offerings. Ideally, within-subjects experiments use counterbalancing to avoid confounding [23]. We used such counterbalancing in our study. Specifically, we developed six clinical scenarios (Appendix), with two scenarios about patients experiencing distress, two scenarios about patients requesting a particular treatment, and two scenarios about patients needing culturally competent care. Three of the six scenarios referred to people living with type 1 diabetes; the other three to people with type 2 diabetes. For each scenario, we presented the scenario in text in the online study and asked participating learners to briefly write in the study web form how they would respond to the situation.

### Deviations from protocol due to Covid-19 restrictions

We had originally planned to conduct in-lab psychometric evaluation of the modules during early 2020 and then proceed to a within-subjects randomized pilot experiment. However, with the declaration of the Covid-19 pandemic in March 2020 and associated public health restrictions at our institution, we were no longer able to finish the partially-complete (*n* = 8 of a planned 30) in-person laboratory evaluations of the modules. We therefore rapidly changed our plan to run an entirely online study by recruiting learners in a course that had suddenly shifted to be delivered online. Our original plan and protocol as of February 28, 2020 is registered at Open Science Framework (osf.io/xpywf), along with the full questionnaire, which was finalized prior to collecting data for the present study (osf.io/5neyw). As outlined in the protocol, we had originally anticipated measuring learners’ empathy as an outcome. However, we were unable to identify a validated scale that was available in French within the rapid time frame of the present study. Additionally, after discussions with patient partners, we determined that how a health professional feels may be less important to a patient than what they say and do. Therefore, in the present study, we focused on structured assessments of learners’ responses to the pre-planned clinical scenarios. Neither these assessments nor the scenarios changed from our original plan.

### Allocation

**Fig. 1:**
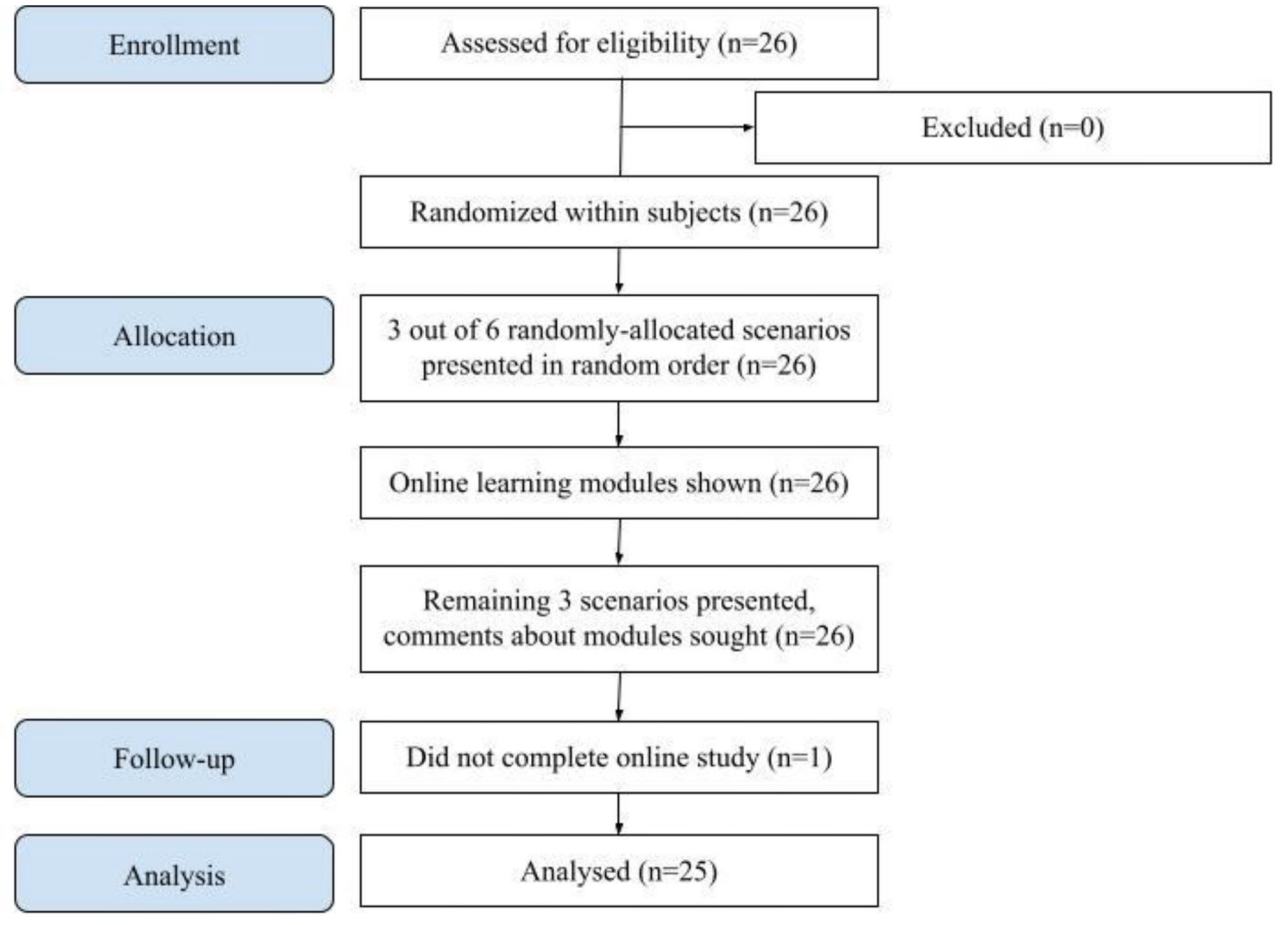
Study flow diagram

Participants saw and responded to three randomly-assigned scenarios prior to viewing the online learning module, and the remaining three scenarios after viewing the module. The randomization was such that each participant saw and responded to one scenario from each of the three topics (patients experiencing distress, patients requesting a particular treatment, patients needing culturally competent care) prior to viewing the online learning modules and the other scenario for that topic after viewing the online learning modules. Within each set of three scenarios, the scenarios were presented in random order. We used Qualtrics (Provo, UT), an online computerized survey software. This software used computerized randomization to automatically assign participants to each predefined sequence. Random allocation helped minimize information and confounding bias. Figure 1 presents the online survey flow, including fixed and random components.

### Allocation concealment mechanism and blinding (masking)

This was a double-blinded study. First, we masked participants to the fact that they were randomized to a particular sequence. Second, we masked investigators to the sequence allocation during data analysis.

### Participants

We conducted this study at one participating Canadian university located in the province of Quebec, Canada. Eligible participants were learners in their final year of pre-clinical study in the medical school of Université Laval. To ensure that participants did not have prior access to similar course content, we chose a group that had not and would not participate in any other patient-oriented research project before or during the intervention. For the experiment, we aimed to recruit at least 10% of approximately 220 medical students registered in the course, which is considered acceptable for pilot experimental studies [24–26]. To be eligible, participants had to: be 18 years old or older at the time of the participation; registered in the aforementioned course in medical school; provide free and informed consent; be able to understand spoken or written French (language of instruction at the institution); and be able to see images and text on a computer screen. For this pilot study, we excluded anyone who could not be exposed to video modules; for example, people living with epilepsy or migraines that could be triggered by cuts between segments in our draft videos.

### Recruitment

In April 2020, we provided patient-led modules to eligible participants through the institutional website. The intervention was an optional activity at the end of an academic course, the fourth of a series of four courses entitled, “Physician, medicine, and society,” (*Médecin, médecine et société* in the original French [27]). The series is delivered one course at a time, spaced across six to nine academic terms. The second and fourth courses in this series explicitly aim to develop learners’ competencies in communication and in the patient-physician relationship. We worked with the course instructors to ensure alignment between the intervention and training expectations. Following ethics approval from Université Laval, participants had up to four months to complete the study. This study was approved by the Ethics Committee of Université Laval (#2016 − 287), including the recruitment announcement, and not giving any incentives for participation in this study. As we had committed to do at the outset of the broader project within which this study is situated, we informed patient partners appearing in the online learning modules of the use of their videos in this particular research project and offered them the opportunity to withdraw their videos if they wished. Course instructors sent the recruitment announcement online only to eligible learners one time in May 2020.

#### Data collection

We collected self-reported demographics (age, race/ethnicity, gender) and whether learners lived with diabetes personally or had someone close to them who lived with a chronic illness. We collected learners’ responses to questions related to three pairs of clinical scenarios (Appendix). We asked all participants to provide responses based on what they would have done if they were the health professionals involved in providing care to these patients. The specific instructions for each scenario were, “Describe your attitude, what you will say and what you will do.” Participants could write as much or as little as they wished. We added optional open-ended questions at the end of the survey to gather information regarding learners’ emotions (“What emotions do these videos evoke in you?”), perceptions regarding diversity (“How do you feel about having people who are different from you (in terms of gender, language, ethnicity, race) in the video modules?”), opinion about video medium versus in-person meetings (“How do you feel about listening to patients by video instead of face-to-face?”) and other comments/advice regarding our teaching modules features (“Do you have any suggestions to improve the message of the videos you’ve seen? Do you have any additional comments?”).

### Data transformation and management

#### Evaluation grid for clinical scenarios

In collaboration with one clinician-teacher (SN), we developed an evaluation grid aligning with the biopsychosocial model integrated in the clinical approach about chronic disease management in medical schools [28]. Our scoring system was consistent with our objective and compatible with academic expectations regarding medical competencies and knowledge and the well-known patient-centered clinical method used in medical schools (Table 1). At Université Laval, students are initiated to the patient-centered clinical method in their first year of medical school. The patient-centered clinical method is often opposed to the disease-centered method focusing only or mostly on scientific explanation of a patient’s illness [29]. Two research assistants independently coded participants’ responses to scenarios by scoring the criteria of the biopsychosocial approach depending on whether they were “discussed,” “partly discussed,” or “non-addressed.” As noted previously, research assistants scored each response without knowing whether participants wrote the response before or after viewing the online learning modules. The same two research assistants assessed all responses.

Based on the recommendation of an evaluation expert (JSR), we gave each criterion an identical weight of 1. Three team members (RN, SN, HOW) discussed and reached consensus on using non-equidistant values for the 3 performance levels (0, 0.6, 1) to better reflect our aim to evaluate whether the videos foster (not create) awareness of the importance of patient-centered care. We also considered the fact that eligible participants would be junior learners with incomplete training on these aspects. Therefore, scores for “partly discussed” criteria (0.6) were closer to “discussed” criteria (1) than to “non-addressed” (0). Satisfactory responses to clinical scenarios were defined as responses yielding an average score of 0.6 or above.

**Table 1:** Evaluation grid

Steps	Description	Insufficient or not discussed	Partly sufficient or partly discussed	Sufficiently discussed
**Step 1** Explore the disease together with the discomfort it causes	History, physical examination, investigation.	The learner does not consider the patient’s disease in their thinking.	The learner somehow takes into consideration the patient’s disease in their reflection.	The learner implicitly or explicitly considers the patient’s disease in their thinking.
	Dimensions of the disease: feelings, ideas, effects on life and expectations.	The learner does not name the patient’s feelings, ideas, effects on life and expectations.	The learner mentioned some of the patient’s feelings, ideas, effects on life and expectations.	The learner implicitly or explicitly considers the patient’s feelings, ideas, effects on life and expectations.
**Step 2** Have a global approach to understanding the person	The person: their life history, personal background and the context of their personal development and values.	The learner does not consider the patient’s individual context.	The learner partly considers the patient’s individual context.	The learner considers the patient’s individual context.
	Individual context: family, employment, social support, finances, education.	The learner does not consider the patient’s individual context.	The learner partly considers the patient’s individual context.	The learner considers the patient’s individual context.
	The global context: culture, community, ecosystem.	The learner does not consider the patient’s social and global context, including their culture and community.	The learner partly considers the patient’s social and global context, including their culture and community.	The learner considers the patient’s social and global context, including their culture and community.
**Step 3** Find a common ground	Problems and priorities; treatment or management goals; patient and physician roles.	The learner does not try to find a common ground. Among other things, he/she does not mention the problems or priorities of the patient and the physician. They do not clarify treatment goals.	The learner tries to find a common ground. Among other things, he/she mentioned some problems **or** priorities of the patient and the physician. They provide little clarification of treatment goals.	The learner tries to find a common ground. Among other things, he/she names the problems **and** priorities of the patient and the physician. They try to clarify the treatment goals.
**Step 4** Incorporate the prevention aspect along with health promotion	Improving health; ability to avoid risks; risk reduction; early identification; reducing complications.	The learner does not suggest any solutions to improve the patient’s health, reduce harm and/or anticipate complications.	The learner implicitly suggests solutions to improve the patient’s health, reduce harm and/or anticipate complications.	The learner suggests solutions to improve the patient’s health, reduce harm and/or anticipate complications.
**Step 5** Improve the physician- patient relationship	Compassion; power; healing; self-awareness; transference and counter- transference.	The learner exhibits no compassion in their statement.	The learner exhibits some compassion in their statement.	The learner exhibits compassion in their statement.

#### Structured responses to open-ended questions

Since it is not recommended to use survey data for in-depth content analyses [30], we planned to organize participants’ responses to open-ended questions using categories while stating the rationale for each of these categories, reporting the number of statements, and providing representative quotes.

We stored original data on Qualtrics servers located in Canada and on a secure local server at the participating university. Only team members with complete and relevant ethics training had access to collected data. Computers used for analyses were protected by a password. All the data collected were confidential and used for research purposes only.

### Outcomes

Our primary outcome was learners’ satisfactory responses to clinical scenarios-related questions answered after versus before the intervention. Our secondary outcome was learners’ feedback about video modules from patients’ narratives.

### Analyses

Descriptive analyses included the number (proportion) of participants by individual characteristics. Participants’ responses to clinical scenarios randomly displayed before and after the intervention were respectively treated as pre-intervention and post-intervention data. For each participant, we added up data separately for each criterion and then calculated pre- and post-intervention average scores by dividing the scores’ summation by the number of criteria. Such a compensatory approach allows stronger performance on some criteria to potentially compensate for weaker performance on others. If we were to use a non-compensatory scoring, failure of one or the other criteria would have resulted in a failure of the entire assessment. To clearly differentiate the effect of the within-subjects experiment, we calculated overall scores by pre- or post-intervention criterion rather than by scenario. Using a score of 0.6 as the cut-off point over which responses were considered as satisfactory (also called passing score), we compared pre-intervention and post-intervention overall scores. We used the Fisher Exact probability test which allows us to compare proportions within a small sample.

## Results

Participants (*n* = 26) were mostly white, non-Indigenous (23) adults and mostly identified as women (20). The proportion of women is approximately reflective of the makeup of the student body of the participating medical school. Two participants self-reported as living with diabetes and ten self-reported as having someone close to them who lives with a chronic disease. As shown in Fig. 1, one person began but did not complete the study, resulting in *n* = 25 complete study responses available for analysis.

In examining satisfactory responses to clinical scenarios in the pre- versus post-intervention data, we observed a significant difference between learners’ scores that were equal or above the passing score of 0.6 (Table 2). While 66% of pre-intervention scores were at or above the passing score prior to the intervention, this percentage was 76% for post-intervention scores. This difference is statistically significant (*p* = 0.002). An improvement of 10 percentage points in acceptable patient-centered care scores is also meaningful from an educational and healthcare quality perspective.

**Table 2:** Percentages of passing scores in response to clinical scenarios

Percentages of scores ≥ 0.6		*p*-value
Mean pre-intervention scores among randomly-assigned 3 scenarios per learner	Mean post-intervention scores among randomly-assigned 3 scenarios per learner	
66%	76%	0.002

In open-ended questions, learners expressed appreciation for the opportunity to hear directly from diverse people with diabetes about diabetes care and management (Table 3).

**Table 3:** Learners’ reactions to patient-led teaching modules

Categories	Definitions	Number of statements	Example of quotes (translated from the original French)
Emotions			
Disappointment Guilt Frustration	Negative feelings in relation to patient-led teaching modules	6	“[…] disappointment regarding to certain experiences that patients must have had” “outrage at my own ignorance of the reality in which patients with diabetes mellitus live”
Empathy Openness	Putting self in the patients’ shoes and understanding what the patients are feeling	11	“It reminds us of the great responsibilities of doctors towards their patients.” “I see how important it is to patients that we take care of the patient in a comprehensive way.” “I notice that diabetes is a much more anxiety-provoking condition than I thought and that you have to make sure you explain it to the patient.”
**Perceptions regarding diversity of expert patients in online learning modules**			
Positive attitude	Openness to the differences of others in the context of future practice as physician	12	“I laughed and got emotional at times. The testimonials of the mothers in particular opened my eyes to this perspective that I had not yet considered: the stress put on the parents of a diabetic child.” “Really very, very nice! Although each person is a unique individual, the more patient archetypes we are able to encounter, the more we can integrate a better understanding of diverse realities [into our future practice].” “Interesting and necessary: ​​it makes the answers more varied.”
Indifference		1	“I didn’t really feel any differences”
Ambivalence	Perceiving both pros and cons or being neutral	1	“No problem…switching from English to French several times detracts from the experience.”
**Opinion about video medium versus in-person meetings**			
Cons	Face-to-face preference	4	“It allows me to have the point of view of the patients. On the other hand, this limits the interaction.”
Pros	Video preference	4	“[It] allows patients to better deliver their message without being intimidated by the presence of strangers (with whom they may not have a relationship of trust.)”
Ambivalence	Perceiving advantages of both medium	3	“The 2 methods are complementary in my opinion.”
Indifference	No preference	2	“Both give the same result.”
Other	Other	1	“I would have preferred to have had a full video and not little bits and pieces each time. It would be more realistic.”
**Technical aspects**			
User perspectives	What may be improved	14	“Avoid repetitions [that use the same video segments in the different modules]”
**Other comments**			
Positive comments	Encouragements	4	“I think it’s very important as a learner to have the patients’ point of view. If we want to improve the profession of medicine, we need to ask them what to work on.” “The testimonials collected included relevant remarks.”

## Discussion

In this study, we aimed to determine whether patient-led online learning modules can influence health professions learners’ responses to relevant clinical scenarios and to collect learners’ feedback about videos. Our findings suggest that viewing online learning modules showing patients’ perspectives about diabetes care and management can significantly improve learners’ responses to clinical scenarios. Our findings also suggested that patient-led teaching modules may have positive impacts on learners’ education. Given that our scoring system closely aligned with the biopsychosocial model [28, 31] and the well-known patient-centered clinical method used in medical schools [29], our findings more broadly suggested that the exposure of health professions learners to patient-led teaching may improve their understanding of what matters to patients in chronic disease care and management.

Learning from patients may help future clinicians bridge the traditional gap between scientific knowledge of chronic diseases and experiential knowledge of how health professionals can help or harm patients’ experiences of living with chronic conditions [32]. Our findings align with previous work showing that exposure to patients’ experiences and suggestions is an effective pedagogical tool in health professions education and has a positive impact on learners’ attitudes, knowledge, and skills [16]. Unlike previous work reflecting on challenges for health professionals to apply patients’ in-person presentations in their practice [33], our study suggests that online learning modules assembled from patients’ video presentations may offer similar pedagogical benefits while being more scalable. More scalable solutions make it easier for more patients to participate and for clinicians to learn at any time.

Learners in our study appreciated having people from diverse ethnicities sharing their perspectives, as it opened their minds regarding how identity characteristics may shape patients’ experiences and satisfaction with care. Both family medicine residents who performed prior analyzes of the teaching modules also mentioned that the listening to patients’ narratives aligned with and reinforced the theoretical teaching they had received in their courses. Their experience suggests that patient-led online learning modules represent an opportunity to reinforce traditional academic training. This is consistent with a previous study suggesting that patients’ perspectives contribute to reinforce learners’ empathy and understanding of patients [34]. 

### Strengths and limitations

This study has five main strengths. First, ten patient partners contributed to the original project that led to this study [20] and helped us make sure that we adequately acknowledged and integrated the subjective nature of patient narratives [1]. Second, learning modules used in the intervention assembled multiple perspectives from diverse patients without compromising their raw and authentic nature [1, 35], which therefore offered a multitude of learning opportunities for learners. Third, using an online medium compared to formal in-person presentations contributed to improving the accessibility of patients’ knowledge and wisdom for learners. Each video in our patient-led teaching module was relatively short (2–3 min), enabling it to be broadcast in multiple sites/groups. Fourth, our within-subject experiment allowed us to assign participating learners to randomized scenarios and questions. This approach represents a practical way to address the accreditation requirement for medical schools to provide and maintain the same training for all learners in a given course while allowing randomized study designs. Fifth and finally, online learning modules consisting of patient-led video narratives are scalable interventions that can be replicated in other chronic disease and health education contexts. From learners’ perspectives, our videos have the potential to foster learning and practice so that future health professionals may provide patient-centered health care.

Our study also has some limitations. First, because the study took place within a medical school curriculum that puts a certain amount of emphasis on communication skills and patient-centered care, it is possible that a similar intervention may have greater or lesser effects within curricula that place more or less emphasis on such competencies. Second, our convenience sample was not representative of all learners in the target population, which has a greater proportion of racialized people and members of ethnocultural minority groups, and our sample size does not allow us to stratify analyses by diabetes types or learners’ individual characteristics. Future studies should consider exploring the contribution of individual characteristics in such interventions. Third, due to public health restrictions, we were unable to complete a planned study prior to the present study to optimize the online learning modules by ensuring they do not cause unduly negative emotions nor cognitive overload [2, 36, 37]. While participants’ responses to open-ended questions at the end of the study did offer ideas for future improvements, such as potentially showing each interview in its entirety rather than grouping portions of videos by theme, responses nonetheless suggested that viewing the videos did not elicit major negative emotions. This may be partly due to the number of participants who self-reported as patients or caregivers of someone with a chronic disease compared to the general population, which may be considered both a strength and a limitation. Fourth and finally, this pilot project did not aim to validate the scenarios and questionnaires, nor evaluate the effects of patient-led online learning modules on learners’ behavior months or years later. It is possible that the effects we observed may be transient or may not translate into improved behavior. Personal stories are often very salient and memorable, but further research is needed to determine whether a 45-minute intervention such as this has lasting effects on future health professionals’ interactions with patients.

### Implications

This pilot study represents an example of how to integrate patients’ perspectives concerning the alignment between chronic disease care and the competencies expected from health professionals in health education. If designed, tested, and disseminated appropriately, video narratives featuring people living with health conditions and challenges may help mitigate the risk of focusing on quantitative data at the expense of the singularity and significance of patients’ narratives [16, 38–40]. Future research may include replicating our study in more health professions programs and institutions, as well as exploring the potential of such interventions to improve knowledge and provision of patient-centered care.

## Conclusion

By leveraging the richness and power of patients’ expertise, our findings suggest that learners in the health professions can learn to provide better chronic disease care by listening to patients about how health professionals can provide better care, and that such listening can be facilitated through online patient-led learning modules.

### Electronic supplementary material

Below is the link to the electronic supplementary material.

Supplementary Material 1

## Data Availability

For ethical reasons, video narratives and learners’ responses are not available to the public. The data will be kept in the principal investigator’s laboratory located on the campus of Université Laval and only members of the research team will have access to data. The data stored on Qualtrics Canadian servers located in Canada are subject to Canadian data protection laws.

## Declarations

### Previous presentations

Ndjaboue R, Chipenda Dansokho S, Tremblay MC, Dogba J, Price R, Delgado P, McComber AM, Boudreault B, Frigon-Tremblay F, Drescher O, McGavock J, Witteman HO. A patient-initiated research project to improve diabetes management and care. Diabetes Action Canada Summer. Moved online due to pandemic. July 29, 2020, Canada.

Ndjaboue R, Dagnault A, Delgado P, Dogba J, Drescher O, Numainville S, Tremblay M-C, Witteman H. (2021). Integrating patients’ voices in health professions education: a pilot randomized controlled trial. 43rd Annual Meeting of the Society for Medical Decision Making. « From the individual to society: how systems and social factors shape health care decisions ». October 18–20, Virtual, Canada.
