# Supplementary material for: Patients as teachers: a within-subjects randomized pilot experiment of patient-led online learning modules for health professionals
Source: BMC Med Educ. 2024 May 10;24:525. doi: 10.1186/s12909-024-05473-4 (PMC11087246; doi:10.1186/s12909-024-05473-4)
Supplement: Supplementary file 1 — Supplementary Material 1 [file 12909_2024_5473_MOESM1_ESM.pdf]

APPENDIX. Clinical scenarios (translated from original French)

Scenario 1: Mrs N. aged 48 years old has a 16-year-old daughter newly diagnosed with type 1 diabetes. You encounter them for the first time. Blood glucose test results in the last two weeks were often higher than target values. History: Diagnosed with type 1 diabetes 4 months ago. Weight 49 kg, height 155 cm. Lab results of the previous week show acceptable figures; including HbA1c 7.1% (54 mmol/mol) and a random blood glucose of 4.0 mmol/L. No family history of diabetes of either type. Reasons for the visit: Mrs. N. wants your opinion regarding her daughter's ability to participate in Ramadan. Ramadan is a religious requirement for people observing Islamic law which requires 40 consecutive days of food deprivation. As usual, the whole family is preparing for Ramadan by gradually reducing nutrient intake. Starting the next day, everyone participating must fast on an empty stomach from sunrise to sunset for 40 days. Describe your attitude, what you will say and do.

Scenario 2: Ms. J. aged 19 years old, has a follow-up medical encounter for her diabetes. You haven't seen her for almost 6 months. Her last two glycemic checks revealed a glycated hemoglobin of 7.1% (54 mmol/mol) 12 months ago and 7.7% (61 mmol/mol) 9 months ago. History: Diagnosed with type 1 diabetes at age 9. Member of the swimming team of her university. Weight 64 kg, height 170 cm. Family history: an older brother with type 1 diabetes diagnosed at the age of 7. Reasons for the visit: The results of the last two laboratory tests (at 6 months and 3 months) are missing from Ms J.'s file because she missed all her appointments despite multiple reminders. When you enter the office, the patient begins to cry bitterly without being able to contain herself and without obvious reason. She experiences psychological distress in relation to difficult times in her personal life. You hesitate between postponing the appointment to another time to give her time to calm down, receiving her briefly to meet the most urgent needs or taking more time to meet. Describe your attitude, what you will say and what you will do.

Scenario 3: You meet Mr. G., aged 49 years old, who made an appointment to discuss medication prescriptions. Mr. G.'s latest blood sugar results sometimes showed high blood sugar numbers, which worries him because he strictly follows the dietary and physical activity recommendations given to him. History: Diagnosed with type 1 diabetes at age 18. Weight 73 kg, height 175 cm, BMI 23.8. Lab test results of that particular day show normal numbers; in particular a glycated hemoglobin 6.2% (44 mmol/mol) and a random blood glucose of 5.6 mmol/L. No family history of diabetes to date. Reason for visit: Mr. G. wants to start a medication with which you are uncomfortable because you do not know enough about this new medication. He tells you that it is Fiasp, a new rapid-acting insulin recently approved in Canada (you do not know if it is on the provincial public assurance form). Since this drug acts more quickly, its risk profile is therefore different for hyper- and hypo-glycaemia. Describe your attitude, what you will say and what you will do.

Scenario 4: During a routine appointment, you meet Mr. T., a 65-year-old Indigenous man. Mr. T has been on insulin for nearly nine months. Mr. T.'s latest blood sugar results showed blood sugar numbers within expected values. He has never missed his appointments, he has a healthy diet from locally harvested products and without fertilizers and pesticides. He also tells you that

he does his best to live a stress-free life. History: Diagnosed with type 2 diabetes at age 35. Weight 69 kg, height 165 cm, BMI: 25.3. Lab test results of the same day show normal numbers; in particular a glycated hemoglobin 6.2% (44 mmol/mol) and a random blood glucose of 5.6 mmol/L. Background: Several community and family members have been diagnosed with type 2 diabetes. Reasons for the visit: Mr. T. wants your opinion concerning his intention to attend a traditional territorial ceremony during which the whole community must remain on an empty stomach from sunrise to sunset. You are uncomfortable with the fact that Mr. T will not take insulin or meals for more than 12 hours. Describe your attitude, what you will say and what you will do.

Scenario 5: Ms. A., 42 years old, an immigrant from French-speaking Africa, has lived in Quebec for 5 years. Following a minor knee accident, she stopped exercising and gained 10 kg in 9 months. You have prescribed specific laboratory tests for further monitoring because she had an abnormal blood glucose value two months ago (7.6 mmol/L fasting glucose). Ms. A. comes in consultation to follow up on the results of her annual visit last week. History: Obesity (weight 90 kg, height 152 cm, BMI: 38.95. Family history: Her mother had similar symptoms and was diagnosed with prediabetes. Reasons for the visit: The results of Ms. A.'s laboratory tests show several out-of-standard values. Specifically, fasting glucose 7.8 mmol/L and glycated hemoglobin 7.3% (56 mmol/mol). Her C-peptide level is normal, indicating that her diabetes is type 2. You are responsible for telling Ms. A. that she has been diagnosed with type 2 diabetes. Ms. A informs you that she has difficulty following the recommendations you gave her for her diet at her last appointment and she experiences a lot of guilt for not being able to stabilize her weight despite the advice you gave her. You will need to discuss with her the relationship between weight loss and type 2 diabetes management. You have 15 minutes to meet with her. Describe your attitude, what you will say and what you will do.

Scenario 6: Mr. R., a 56-year-old Indigenous man, comes to the clinic because his blood sugar levels are high. His last three glycemic checks were 10.6 mmol/L, 7.7 mmol/L and 8.9 mmol/L. Glycated hemoglobin 8.3% (67 mmol/mol). History: Mild obesity (weight 92 kg, height 172 cm, BMI 31.1; Type 2 diabetes for 11 years, treated with metformin 850 mg ibid (2/day); hypertension treated and well-controlled for 7 years. Family history of obesity and diabetes: father and one sister. Reasons for the visit: Mr. R. mentions that he does not have enough money to eat healthfully. In speaking with you, he tells you that he has challenges eating healthy food because he does not have enough income to buy it. Mr. R. needs a medical professional to sign a form allowing him to obtain financial assistance to buy food. Describe your attitude, what you will say and what you will do.
